# Unravelling how low dominance in faces biases non-spatial attention

**DOI:** 10.1038/s41598-019-54295-8

**Published:** 2019-11-29

**Authors:** Ashton Roberts, Romina Palermo, Troy A. W. Visser

**Affiliations:** 1 School of Psychological Science, The University of Western Australia, Perth, Western Australia Australia; 2 ARC Centre of Excellence in Cognition and its Disorders (CCD), Sydney, Australia

**Keywords:** Psychology, Human behaviour

## Abstract

According to the Dual Model of Social Hierarchy, one pathway for attaining social status is through dominance (coercion and intimidation). High dominance stimuli are known to more readily attract eye gaze and social attention. However, when there is a competition for non-spatial attentional resources, low dominance stimuli show an advantage. This low dominance bias was hypothesised to occur due to either counter-stereotypicality or attention competition. Here, these two hypotheses were examined across two experiments using modified versions of the attentional blink paradigm, used to measure non-spatial attention, and manipulations of facial dominance in both males and females. The results support the attention competition theory, suggesting that low dominance stimuli have a consistently strong ability to compete for attentional resources. Unexpectedly, high dominance stimuli fluctuate between having a strong and weak ability to compete for the same resources. The results challenge the current understanding of how humans interact with status.

## Introduction

Social hierarchies are common throughout both animal and human societies^1–4^, and typically depend on individuals’ perceived ranking on “valued dimensions”^5,6^. Mattan, Kubota, and Cloutier^7^ argued that these “social status” rankings are acquired through knowledge-based and/or perceptual antecedents. These antecedents allow differentiation of status that then leads to a variety of social, cognitive, and behavioural consequences^6,8–11^ – such as deference of decision making responsibilities^12^. The impact, or effectiveness, of the antecedents are modulated by the social context in which they exist. For example, physical strength may be a determinant of status on an Australian Rules Football field, but may be less important in an academic environment.

A prominent explanation for the impact of social status is the Dual Model of Social Hierarchy^6^. According to this model, social status is acquired through either prestige or dominance based pathways. Prestige-based status is an intrinsically human form of status (although see Kendal *et al*.^13^) based on social and knowledge related antecedents such as skill or respect^6,14,15^. Prestige-based status is strongly linked to social learning benefits^16,17^ (see Cheng & Tracy^18^ for a review). By comparison, dominance-based status is associated with intimidation, manipulation, and control over resources^18–23^ – see Cheng *et al*.^14^ for a review of specific dominance-based status associations. Dominance is considered an evolutionary pathway, given its existence in a number of non-human species^24^. The antecedents for dominance are typically readily perceivable physical indicators such as masculinised and mature facial features^25–27^ and differences in voice pitch modulations^28^.

A number of studies have examined how observers’ perceptions are biased by attributes associated with dominance-based status. Studies of gaze frequency and gaze cueing have suggested that people have an attentional bias towards high dominance-based status^14,29,30^. In a typical gaze cueing paradigm (e.g. Driver *et al*.^31^) the gaze of a centrally presented face is directed to the periphery (typically either left or right), after which a target is presented either on the left or the right of the face. Typically, peripheral targets that are aligned with the gaze of the centrally presented face are responded to faster and more accurately than a non-aligned peripheral target^31^. Using masculinised (most dominant) and feminised (least dominant) faces, Jones *et al*.^29^ found that high dominance faces yielded a significant cueing effect at shorter durations. This indicated an involuntary response bias towards high dominance faces, and the effect was replicated in Ohlsen, van Zoest, and van Vugt^32^. Similarly, in the second of two studies, Cheng *et al*.^14^ demonstrated that high-dominance individuals were fixated more often and for longer than their low dominance counterparts.

On the other hand, when stimuli are already at the focus of gaze, there appears to be processing biases towards low rather than high dominance faces. For example, Stewart *et al*.^33^ demonstrated a perceptual advantage for low dominance faces when using a Continuous Flash Suppression paradigm. In this paradigm, a dynamic noise pattern (10hz) was presented to the participants’ non-dominant eye, while a face (varying in facial dominance) was presented at the same time to the dominant eye. The speed in which a participant overcomes the noise pattern suppression to respond to the face indicates a preconscious processing bias. Stewart and colleagues found that low dominance faces overcame the noise pattern suppression faster than high dominance faces, suggesting people may have a bias towards processing low dominance faces faster.

In a more direct test of this question, Roberts, Palermo, and Visser^34^ used an attentional blink paradigm to measure non-spatial attention^35–38^. In the AB paradigm, identification of a second target, within a rapid serial visual presentation (RSVP^39^) of non-target distractors presented at the same location in space, is significantly reduced if presented closely after the first target (especially after 300–400ms^35,37^). This effect, called the attentional blink (AB), is theorised to be the result of a competition for attentional resources between the first and second targets^40^, and can be reduced if the second target is motivationally or emotionally salient^41–43^.

In the Roberts *et al*.^34^ study, researchers presented male faces as the second target (T2) – varying in either dominance, as rated in the Australasian first impression rating scale^44^, or prestige, manipulated by pairing the faces with curriculum vitae (CVs) varying in levels of education attainment^8^. Importantly, at the nadir of the AB (~300–400 ms^36^), participants were significantly more accurate at identifying low dominance target faces compared to neutral or high dominance target faces, irrespective of prestige status manipulations.

To account for this low dominance bias, Roberts *et al*.^34^ suggested two potential explanations. One explanation was couched in terms of counter-stereotypicality, which refers to stimuli that are incongruent with the typical stereotype. Past studies have found that counter-stereotypical stimuli (e.g. male nurses) are perceived more negatively than stereotypical stimuli (e.g. female nurses^45,46^). This is also true for perceptions of dominance. Sutherland, Young, Mootz, and Oldmeadow^47^ asked participants to write spontaneous impressions of male and female faces varying in facial dominance. The authors found that most extreme counter-stereotypical dominance faces (i.e. high dominance female) were evaluated significantly more negatively than their typically stereotyped counterparts.

Critically, these incongruent stimuli also preferentially attract attention^48,49^. For example, Wu and colleagues^49^ used a go/no go task to probe behavioural and electrophysiological responses (event-related potentials; ERPs) to counter-stereotypical items. Within the go/no go task, participants were shown word pairings; *mother* or *other* were paired with either *good* or *bad* words, and within each block were told to respond only if a certain word appeared (i.e. only respond if you see *mother* or *good*). The authors found that *mother* elicited a greater neural response than *other*, and that the neural response amplitude was greater for *mother* + *bad* condition (counter-stereotypical) compared to the *mother* + *good* (stereotypical) condition. Taken together, this counter-stereotype research suggests an explanation for the low dominance bias found by Roberts *et al*.^34^ – stimuli that are incongruent with the stereotype (i.e. low dominance male faces) are allocated proportionally greater attentional resources than stimuli that are congruent with the stereotype (i.e. high dominance male faces).

A second possibility proposed by Roberts *et al*. was based on mechanisms of non-spatial attention competition. Theories that attribute the AB phenomenon to competition for attentional resources between the first target (T1) and the second target (T2) imply that the AB may be considered as an index of the ability of T2 to attract attentional resources in the face of competing attentional demands posed by T1. Based on this notion, Roberts *et al*. proposed that low dominance faces may have a greater ability to compete for attentional resources, thus leading to more accurate identification than for high and neutral dominance T2 faces. Importantly, this explanation does not countermand results in the context of earlier visual search tasks. In this explanation, dominance stimuli are competing with other attentional demands, compared to social attention tasks that present single stimuli at unpredictable spatial locations that are competing to capture the focus of spatial attention, in which high dominance faces have an advantage^14,21,50,51^. As a third possibility, both explanations could be operating at the same time.

The following two experiments examine whether the two explanations posed in Roberts *et al*.^34^ aid in understanding the low dominance bias of non-spatial attention. Both experiments will use modified versions of the AB task used in Roberts *et al*. to test each explanation: counter-stereotypicality and attention competition.

## Experiment 1

The purpose of Experiment 1 was to test whether the advantage for low dominance faces found in Roberts *et al*.^34^ could be explained by the counter-stereotypicality account. To do this, we presented female faces that varied in dominance as the second target in a paradigm identical to that of Roberts *et al*. Unlike low dominance males faces of Roberts *et al*., high dominance female faces were incongruent to stereotypical female stimuli (Sutherland *et al*.^47^). Thus, if the accuracy advantage for low dominance faces in the AB seen by Roberts *et al*. was due to the counter-stereotypicality of male low dominance faces, then we would expect to find an advantage for high dominance female faces in Experiment 1. In order to directly measure counter-stereotypicality of both female and male dominance, participants also completed a counter-stereotypicality questionnaire (Sutherland *et al*.^47^) consisting of female and male faces.

## Method

### Participants

Forty-seven participants (*M* = 21.32, *SD* = 6.02, 14 male, 33 female) were recruited from an introductory psychology unit at the University of Western Australia. All participants reported normal or corrected-to-normal vision, and received partial course credit for their involvement.

### Ethical approval and informed consent

This study was approved under the University of Western Australia Human Research Ethics Board (RA/4/1/8640). The experiment was conducted in accordance with the relevant guidelines and regulations, and all participants signed informed consent documents upon commencing the protocol.

### AB task stimuli

**Figure 1:**
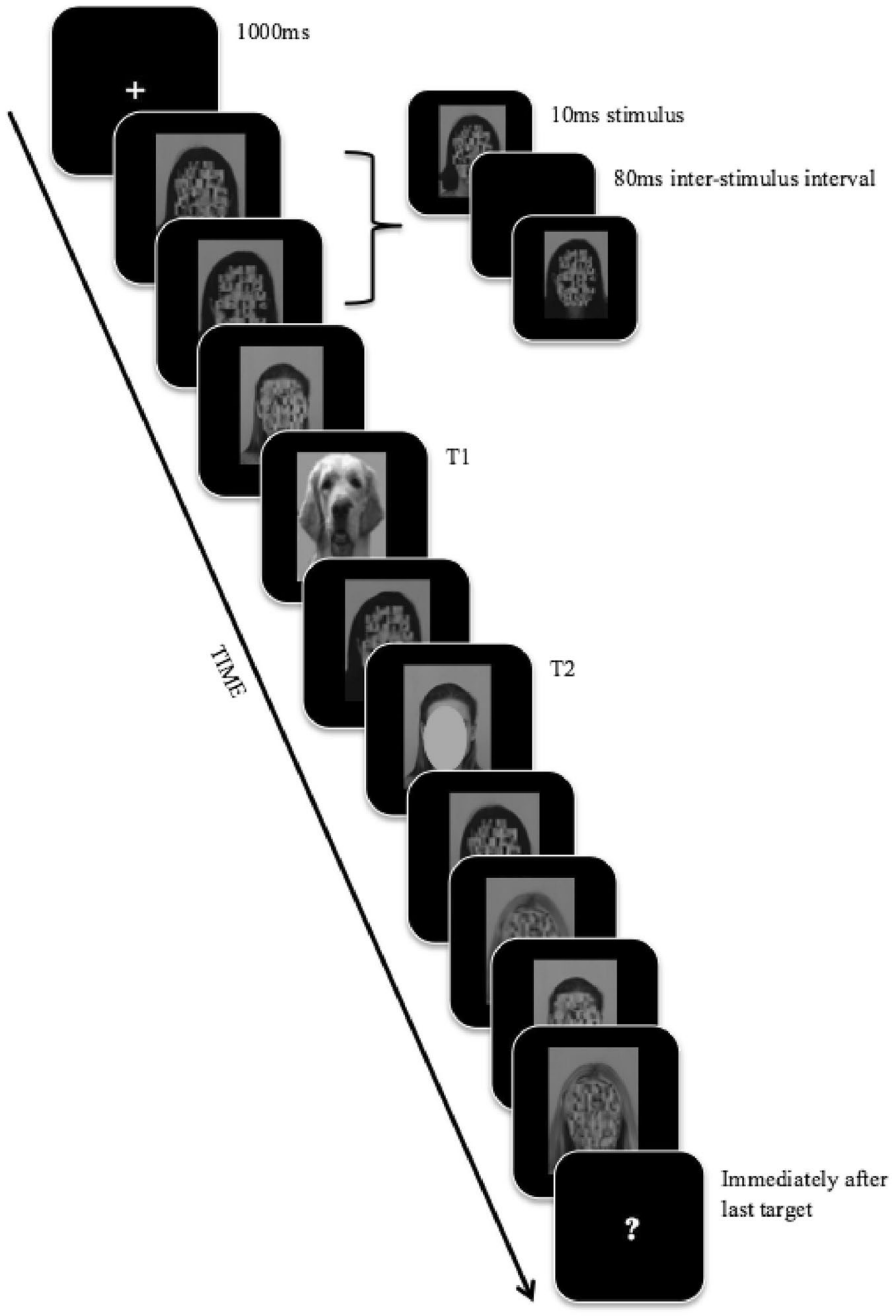
Schematic of the RSVP (lag 2 condition) in Experiment 1. Each stimuli is presented for 10 ms with an inter-stimulus interval of 80 ms – an example is presented at the top of the figure. The schematic is not to scale – stimuli are disproportionally enlarged to show detail. The figure depicts examples of the scrambled, T1 and T2 stimuli. T2 stimulus is hidden in Fig. 1 to protect the individual’s identity.

Six target faces were selected from the Karlolinska Directed Emotional Faces Database (KDEF;^52^; http://tlab.princeton.edu/databases) and were used as the second of two targets (T1 and T2 respectively) in the AB task. Each face was female with a forward facing neutral expression chosen to avoid any emotional expression bias^53^. The faces varied in dominance, as measured using a seven-point first impression rating scale in an Australian sample^26,27,44^. Two high dominance faces (mean rating 4.97/7), two neutral dominance faces (mean rating 4.07/7), and two low dominance faces (mean rating 3.19/7) were chosen. The faces were converted to grey scale, reduced in size (W: 250px, H: 350px), and edited to ensure all faces had similar head sizes, lighting, and eyes at the same level. As in Roberts *et al*., T1 stimuli consisted of two generic dog faces (drawn from English *et al*.^54^). Distractor stimuli consisted of eighteen female faces with neutral expressions that had scrambled facial features (see Fig. 1), also drawn from English *et al*.^54^.

### Counter-Stereotypicality Questionnaire

A counter-stereotypicality questionnaire (CSQ), similar to that used by Sutherland *et al*.^47^, was also administered in order to verify our assumptions about perceived counter-stereotypicality. Participants were shown 12 faces, one at a time, with a blank text box. Six faces were the female faces used in the AB task (high, neutral, and low dominance). In order to have a direct test of counter-stereotypicality seen in Roberts *et al*., the other six faces were males varying in dominance (high, neutral, and low) used in Experiment 2 from Roberts *et al*. Male faces were edited in the same manner as female faces. Participants were instructed to type anything that came to mind when viewing the face, no matter how silly, judgemental, or socially inappropriate. Participants were reminded that all responses were anonymous, that there were no right or wrong answers, and were encouraged to respond to the faces honestly. Participants were told to write as much as they wanted until they felt they were no longer being spontaneous. The CSQ was administered online using Qualtrics software (Qualtrics, Inc. http://www.qualtrics.com/).

### General procedure

E-Prime Psychology Software (E-Prime 2.0; www.pstnet.com/eprime.cfm) was used to create the task, present the stimuli, and collect responses. Participants were seated approximately 60 cm away from a 24” BenQ LCD Monitor (100htz refresh rate). The sequence of events on a typical trial can be seen in Fig. 1. Each trial began with a fixation cross presented for 1000 ms in the centre of the screen (size 18, Courier New font, RGB: 255, 255, 255). This was followed by Rapid Serial Visual Presentation (RSVP) stream of successive images presented at a stimulus-onset asynchrony of 90 ms (10 ms stimulus, 80 ms blank inter-stimulus interval). Four distractor stimuli were presented before T1. Following this, one (lag 2), three (lag 4), or seven (lag 8) distractor stimuli were presented before T2, after which four more distractor stimuli were presented. Following the final distractor image, two successive questions were presented on separate screens. The first question was “Did you see a picture of a dog?” (yes/no). Participants had five seconds to respond using one of two marked keys. The second question was “Which face did you see?” Participants were asked to press a marked key to choose from images of a high, low, or neutral dominance face or a fourth option labelled “No face present”.

Prior to starting the AB task, participants were instructed to identify the dog and the face in each stream of items and to ignore all other items. Participants completed two blocks of trials. Each block consisted of 168 trials divided equally amongst high, low, and neutral dominance T2 stimuli, presented at each of the three lags. In addition, to ensure participants were maintaining consistent observation of the stimuli, there were three types of “catch” trials in each block: trials with no T1, trials with no T2, and trials with no T1 or T2. Each catch trial was presented nine times per lag within each block for a total of 27 catch trials. Thus, in total, participants completed 195 trials per block (390 trials overall). Following the AB task, participants completed the CSQ.

## Results and Discussion

Data from five participants were omitted from the main analyses. Data from two participants were omitted because their overall T1 and T2 accuracy was more than 2.5 *SD* below the mean, while data from three other participants were omitted because their “catch” trials accuracy was more than 2.5 *SD* below the mean. As a result, 42 participants were included in the final analyses (*M* = 21.247, *SD* = 5.86, 11 male, 31 female). Results were analysed with outliers winsorized rather than excluded^55^. The results were comparable, and all conclusions drawn from the analyses remained the same.

### T1 Accuracy

Mean T1 accuracy is shown in Table 1, separated by T1-T2 lag and dominance. A 3 (Dominance: high, neutral, low) x 3 (Lag: 2, 4, 8) Repeated Measures Analysis of Variance (ANOVA) on these means yielded no significant main effect of Dominance or Dominance x Lag interaction effect (*p* > 0.175, *η*^2^_partial_ < 0.038). However there was a main effect of Lag – *F*(2, 82) = 3.57, *p* = 0.033, *η*^2^_partial_ = 0.080, with performance greatest at Lag 4 and poorest at Lag 2. Given the overall high level of accuracy, however, we ascribe no clear interpretation to this finding.

**Table 1:** T1 accuracy as a function of lag and dominance.

	Lag 2	Lag 4	Lag 8
	*M* (*SD*)	*M* (*SD*)	*M* (*SD*)
High Dominance	93.12 (6.07)	95.83 (5.07)	94.51 (5.55)
Neutral Dominance	94.44 (5.72)	95.88 (3.40)	94.08 (4.98)
Low Dominance	93.45 (7.43)	93.68 (7.00)	94.86 (5.09)

### T2|T1 Accuracy

**Figure 2:**
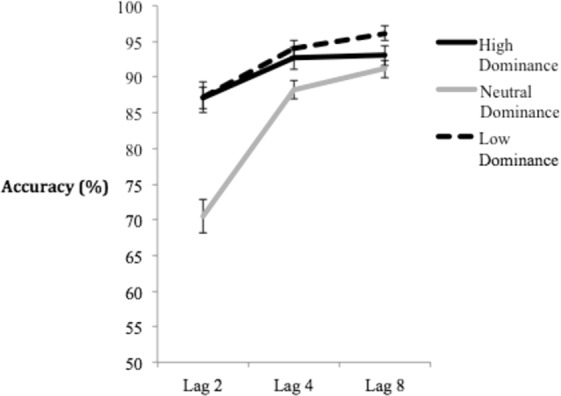
Mean accuracy of the high, low, and neutral dominance conditions across lags 2, 4, and 8. Within-subjects error bars depict 95% confidence intervals, based on O’Brien & Cousineau^79^.

Mean T2 accuracy was calculated only on trials in which participants responded correctly to T1 in order to ensure that they had attended to the first target^37^. These means, separated as a function of dominance and lag can be seen in Fig. 2. A 3 (Dominance) x 3 (Lag) Repeated Measures ANOVA (Greenhouse-Geisser corrected) yielded a statistically significant main effect of Lag, *F*(1.29, 52.90) = 27.21, *p* < 0.001, *η*^2^_partial_ = 0.399, indicating a robust AB, and Dominance, *F*(2, 82) = 26.46, *p* < 0.001, *η*^2^_partial_ = 0.392, indicating high and low dominance faces were generally responded to more accurately than neutral dominance faces. The analysis also revealed a significant Dominance x Lag interaction, *F*(3.12, 127.89) = 12.52, *p* < 0.001, *η*^2^_partial_ = 0.234.

In order to probe the source of this interaction, follow-up analyses were conducted separately at each lag to compare T2 accuracy across levels of dominance. A one-way ANOVA at Lag 2 was significant – *F*(1.77, 72.50) = 31.02, *p* < 0.001, *η*^2^_partial_ = 0.431. Fisher’s LSD post-hoc analyses indicated significant accuracy differences only between neutral dominance and both high dominance (*p* < 0.001, Cohen’s *D* = 0.96) and low dominance (*p* < 0.001, Cohen’s *D* = 0.86) faces. An identical one-way ANOVA at Lag 4 was also significant, *F*(2, 82) = 6.14, *p* = 0.003, *η*^2^_partial_ = 0.130. Fisher’s LSD post-hoc analyses indicated only significant accuracy differences between neutral dominance and both low dominance (*p* = 0.001, Cohen’s *D* = 0.43) and high dominance (*p* = 0.017, Cohen’s *D* = 0.57) faces. These results indicate that the primary interaction effect occurs as a result of high and low dominance faces having a significantly greater ability at competing for attentional resources compared to neutral dominance. Finally, an identical one-way ANOVA at Lag 8 was also significant – *F*(2, 82) = 5.70, *p* = 0.005, *η*^2^_partial_ = 0.122. Fisher’s LSD post-hoc analyses revealed significant accuracy differences only between low dominance and both high dominance (*p* = 0.034, Cohen’s *D* = 0.41) and neutral dominance (*p* = 0.001, Cohen’s *D* = 0.62) faces. This is similar to numerical trends seen in Roberts *et al*. (2019) and suggest that low dominance faces may have a slight perceptual advantage at late lags. However, such an interpretation should be taken cautiously given performance was close to ceiling levels of accuracy (>90%) at Lag 8, and thus difference could be attributable to chance variation^56^.

The results indicate there was a consistent bias towards high and low dominance female faces relative to neutral faces across both shorter lags in the AB task. This is unlike the results of Roberts *et al*.^34^, which demonstrated only a low dominance bias in non-spatial attention. Thus, the current results are inconsistent with the counter-stereotypicality explanation offered by Roberts *et al*. to explain their results.

### CSQ thematic and content analysis

Participant’s responses to the CSQ were analysed following the procedure of Sutherland *et al*.^47^. First, responses for the male and female faces were split into units of single words or short phrases (≤5 words). Words or phrases were not included in circumstances in which shortening or taking then out of context would be misleading. In total, participants wrote 6206 words, which were then separated into 1213 individual words or phrases. On average, participants wrote 2.40 words for each face (*SD* = 1.24, range = 1–7).

**Figure 3:**
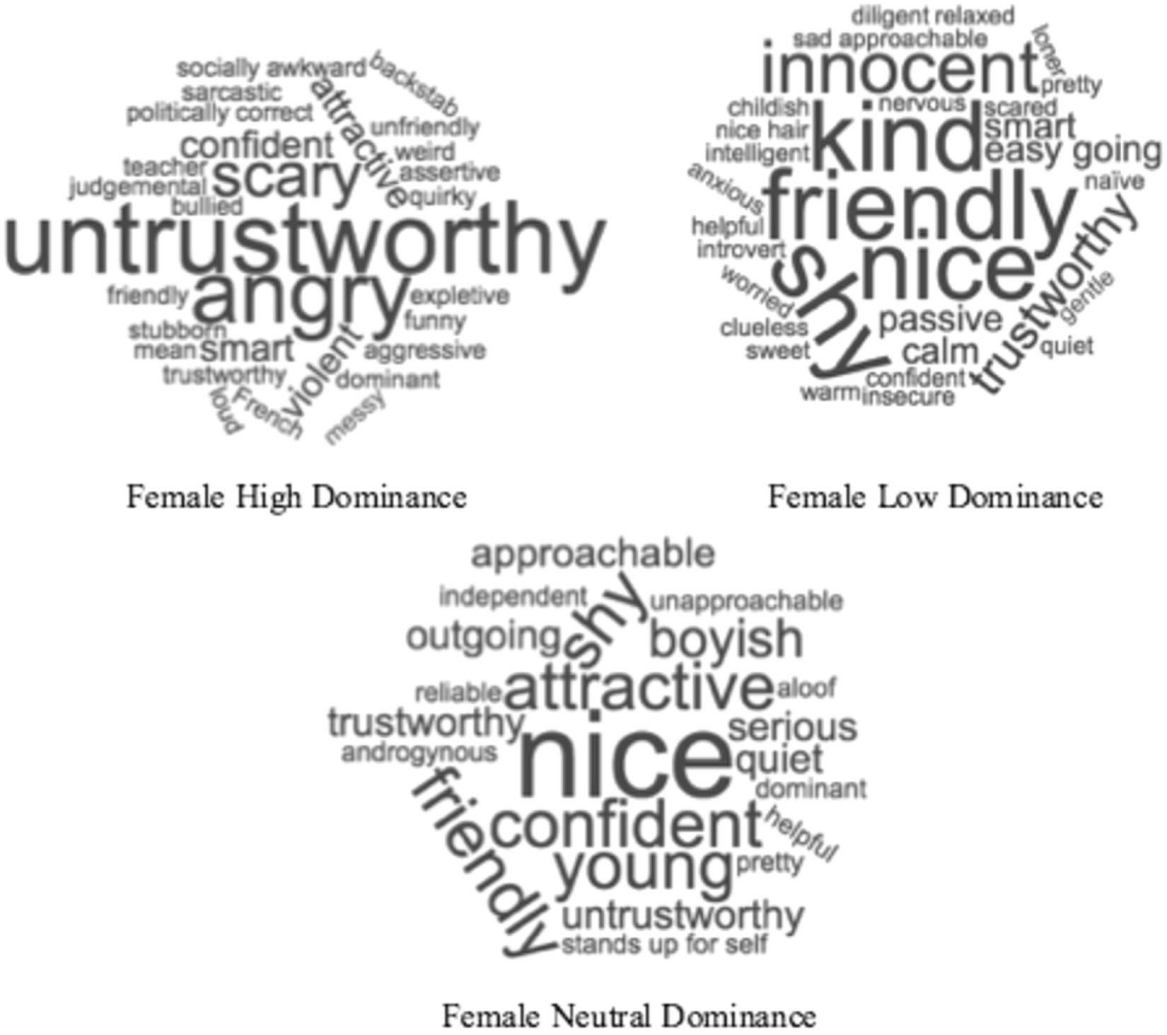
CSQ ratings for female faces varying in dominance (high, neutral, and low). Only descriptions used more than once were included in figure, and larger words indicate higher frequency of use.

**Figure 4:**
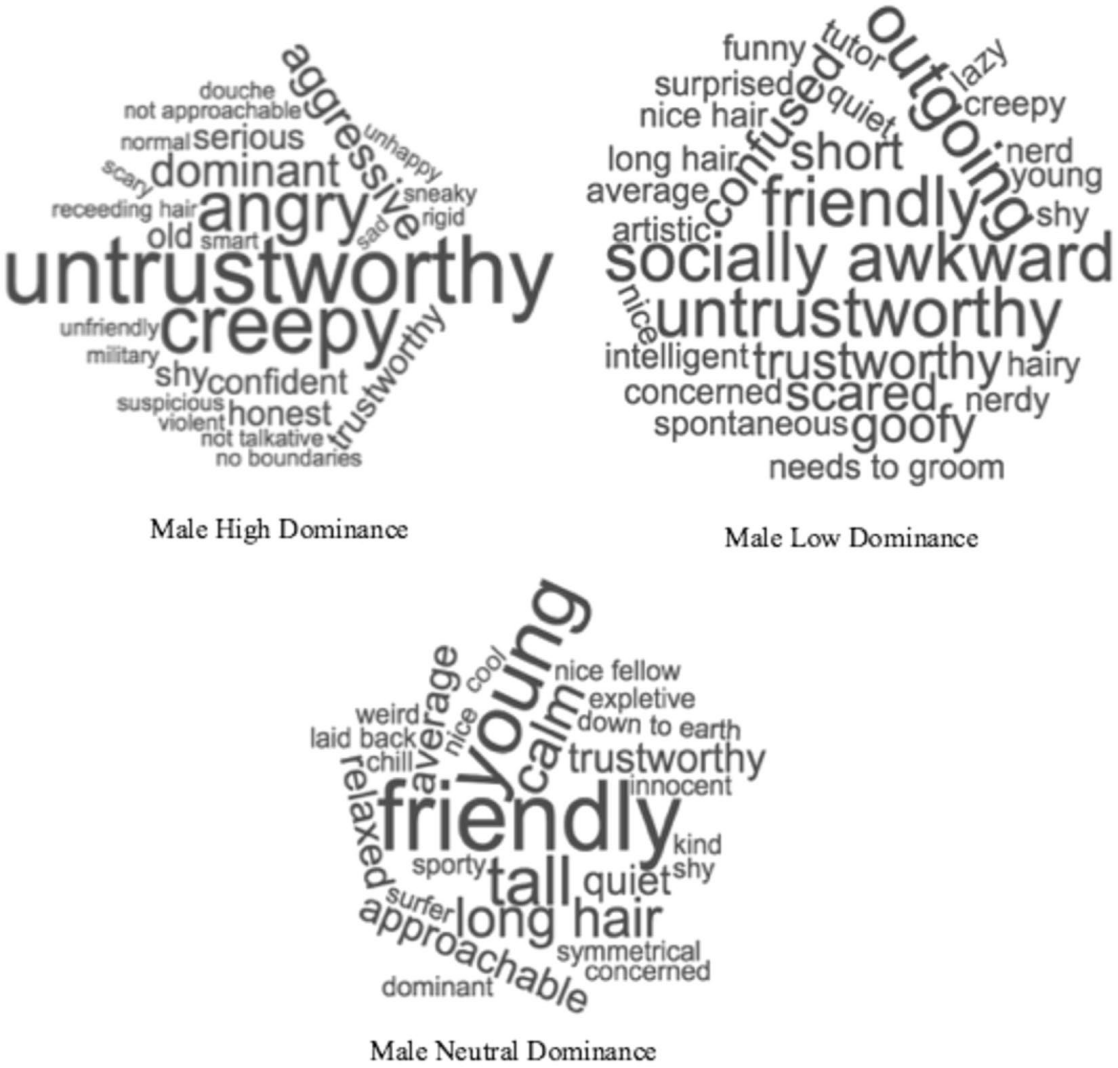
CSQ ratings for male faces varying in dominance (high, neutral, and low). Only descriptions used more than once were included in figure, and larger words indicate higher frequency of use.

Word clouds (https://www.wordclouds.com/) were created to examine words and phrases that were mentioned more than once for each face, with larger words indicating higher frequency of use. As can be seen in Figs. 3 and 4, both male and female neutral faces tended to attract the greatest number of positive responses, with common terms including “friendly” and “nice”. In contrast, the descriptions for both male and female high dominance faces were resoundingly negative such as “untrustworthy” and “creepy”. Low dominance faces had a mixed collection of positive and negative descriptions, such as “friendly” and “nice” for female low dominance, and “friendly” and “socially awkward” for male low dominance.

In order to complement these qualitative observations, a quantitative content analysis was conducted to examine the valence of the spontaneous impressions. Each word or phrase was blind coded by three judges for their *valence* (positive, neutral, or negative). Agreement was high between the judges, as measured by interclass correlation coefficient (ICC = 0.98, p < 0.001), and any disagreements between the judges were agreed upon before the analysis reported below. As with Sutherland *et al*., descriptions were coded as positive, neutral, or negative if they referred to a skill or problem (e.g. “scholar” or “needs to exercise”), used a positive or negative qualifier (e.g. “outstanding face” or “unfortunate looking”), or were conventionally positive or negative (e.g. “friendly” or “unfriendly”). Table 2 below depicts the percentage of positive, neutral, and negative descriptions the male and female faces.

**Table 2:** Percentages of positive, neutral, and negative descriptions for the male and female faces (varying in dominance: high, neutral, and low).

		Positive	Neutral	Negative	Number of Descriptors
Female	High Dominance	25.93	23.61	50.46	216
	Neutral Dominance	45.55	27.75	26.70	191
	Low Dominance	42.92	25.66	31.42	226
Male	High Dominance	18.85	25.13	56.02	191
	Neutral Dominance	36.44	36.44	27.11	225
	Low Dominance	25.13	29.32	45.55	191
					Total = 1240

Descriptions for each participant were coded as follows: a positive description was given +1, neutral was given a 0, and negative was given a −1. For each face, participants’ descriptions were summed to create a valence score. For example, if a participant gave two positives and a negative description for a face, their valence for that face would be 1. Table 3 depicts the average valence for the female and male faces varying in dominance.

**Table 3:** Valence averages (SD) of the male and female faces for each dominance level.

	High Dominance	Neutral Dominance	Low Dominance
Male Faces	−0.75 (1.04)	0.26 (1.16)	−0.38 (0.95)
Female Faces	−0.56 (1.06)	0.42 (1.03)	0.38 (1.24)

*N* = 42.

To examine differences in valence between male and female faces varying in dominance, a 2 (Gender) x 3 (Dominance) Repeated Measures ANOVA was conducted. The ANOVA yielded a significant main effect of Gender – *F*(1, 41) = 7.08, *p* = 0.011, *η*^2^_partial_ = 0.147, which (as can be seen from Table 3) indicates that male faces were rated significantly more negatively than female faces. The ANOVA also yielded a significant main effect of Dominance – *F*(2, 82) = 22.47, *p* < 0.001, *η*^2^_partial_ = 0.354, but no significant interaction effect - *F*(2, 82) = 2.38, *p* = 0.099, *η*^2^_partial_ = 0.055. The lack of a significant interaction effect indicates that the counter-stereotypicality effects are inconsistent with those reported by Sutherland *et al*.

Finally, to further confirm that the counter-stereotypicality account does not provide an adequate explanation for the low dominance bias, a correlation analysis was conducted between the CSQ data and the AB data. Specifically, the magnitude of the AB (i.e. difference between Lag 2 and 4) for low, neutral, and high dominance was calculated and compared with the corresponding valence scores from the CSQ data (e.g. high dominance AB magnitude was compared with high dominance CSQ valence scores). The analyses resulted in non-significant correlations between the AB magnitudes and the CSQ data (*r* = −0.26 – 0.07, *p* = 0.096 – 0.761), demonstrating a lack of connection between the AB results and the CSQ results.

The aim of Experiment 1 was to test the explanation that the bias towards low dominance faces found by Roberts *et al*. could be due to attention to incongruent stimuli; i.e. the counter-stereotypicality of low dominance faces^47^. On this account, it would be expected that *high dominance* female faces would show accuracy advantages in the AB task and be rated more negatively by participants than neutral or low dominance female faces. However, while there was evidence that high dominance females were significantly more negatively rated, both high and low dominance female faces showed a smaller AB than neutral dominance female faces. This suggests that when the results of the CSQ data are examined in relation to the AB data, the counter-stereotypicality argument does not easily account for the findings in Roberts *et al*.

It was hypothesised, based on Sutherland *et al*., that *only* low dominance male faces and high dominance female faces would attract negative responses on the CSQ. However the results found that high dominance male faces also attracted a significant proportion of negative descriptions. This could be due to key features of dominance-based status. As reviewed in previous work (see^6,7,14^), features of dominance-based status are typically associated with intimidation, manipulation, and control over resources – generally negative attributes when compared to prestige based status. However, having both low and high male dominance faces rated negatively has implications for understanding attention to dominance. Depending on the study, both low and high dominance has been found to bias different forms of attention^14,29,33–35^. The implication, therefore, is that negative CSQ responses towards the extremes of dominance may be associated with greater attention. It should be noted that the raters in the current study are not the same as the participants in Roberts *et al*., and this implication is, therefore, merely speculative.

Similarly, the findings from the AB paradigm in the current study show that there was a consistent bias towards high and low dominance female faces relative to neutral faces. In addition to the consistent bias towards low dominance faces seen across genders, the present results also showed a bias towards high dominance female faces, not seen with male faces. This may reflect the fact that switching from male to female facial stimuli might have more complex effects than just a shift in the counter-stereotypical target. For example, there are a number of alternative facial constructs that are both easily recognisable and valued in female faces compared to dominance (such as trustworthiness or youthful attractiveness^26,57^). It may be the case that in the absence of differences in trustworthiness and attractiveness, female face stimuli that reflect the extremes of dominance will bias attention compared to the more common neutral dominance. Further work is needed to understand the effect of counter-stereotypicality, as well as exploring non-spatial attentional biases to female faces

Overall three key findings emerged from Experiment 1. Firstly, the CSQ data suggests that the counter-stereotypicality argument cannot account for the low dominance bias found in Roberts *et al*. Secondly, low dominance female faces appear to significantly bias non-spatial attention – similar to the low dominance bias to male faces seen in Roberts *et al*. Finally, however, Experiment 1 also demonstrated a non-spatial attentional bias towards high dominance female faces, which suggests that there may be significant differences in how attention is allocated to dominant males and females. As previously discussed, there are a number of alternative facial constructs that are valued and easily recognised in female faces, such as trustworthiness or attractiveness^26,57^ – see Zebrowitz and Montepare^58^ for a review. When measured using eye tracking, DeWall and Maner^50^ demonstrated that participants allocated significantly more visual attention to high status males compared to females. Moreover, they found that women who were considered physically attractive captured spatial attention. In short, there may have been a number of other factors, other than counter-stereotypicality, that resulted in biases in favour of both low and high dominance female faces in Experiment 1.

## Experiment 2

Experiment 2 examined whether the advantage for low dominance faces found in Roberts *et al*.^34^ can be explained by their superior ability to compete for attentional resources with other attended stimuli. If this is the case, then it would be expected that low dominance faces inserted as T1 in an AB task would yield a larger AB relative to neutral or high dominance faces, reflecting their proportional advantage over T2 in competition for attention. To test this prediction, we used a version of the AB task employed by Roberts *et al*. (Experiment 2) with male faces varying in dominance as T1, and digits as T2.

## Method

### Participants

Forty-four participants (*M* = 22.27, *SD* = 7.25, 14 male, 30 female) were recruited from an introductory psychology unit at the University of Western Australia. All participants reported normal or corrected-to-normal vision, and received partial course credit for their involvement.

### Materials and procedure

**Figure 5:**
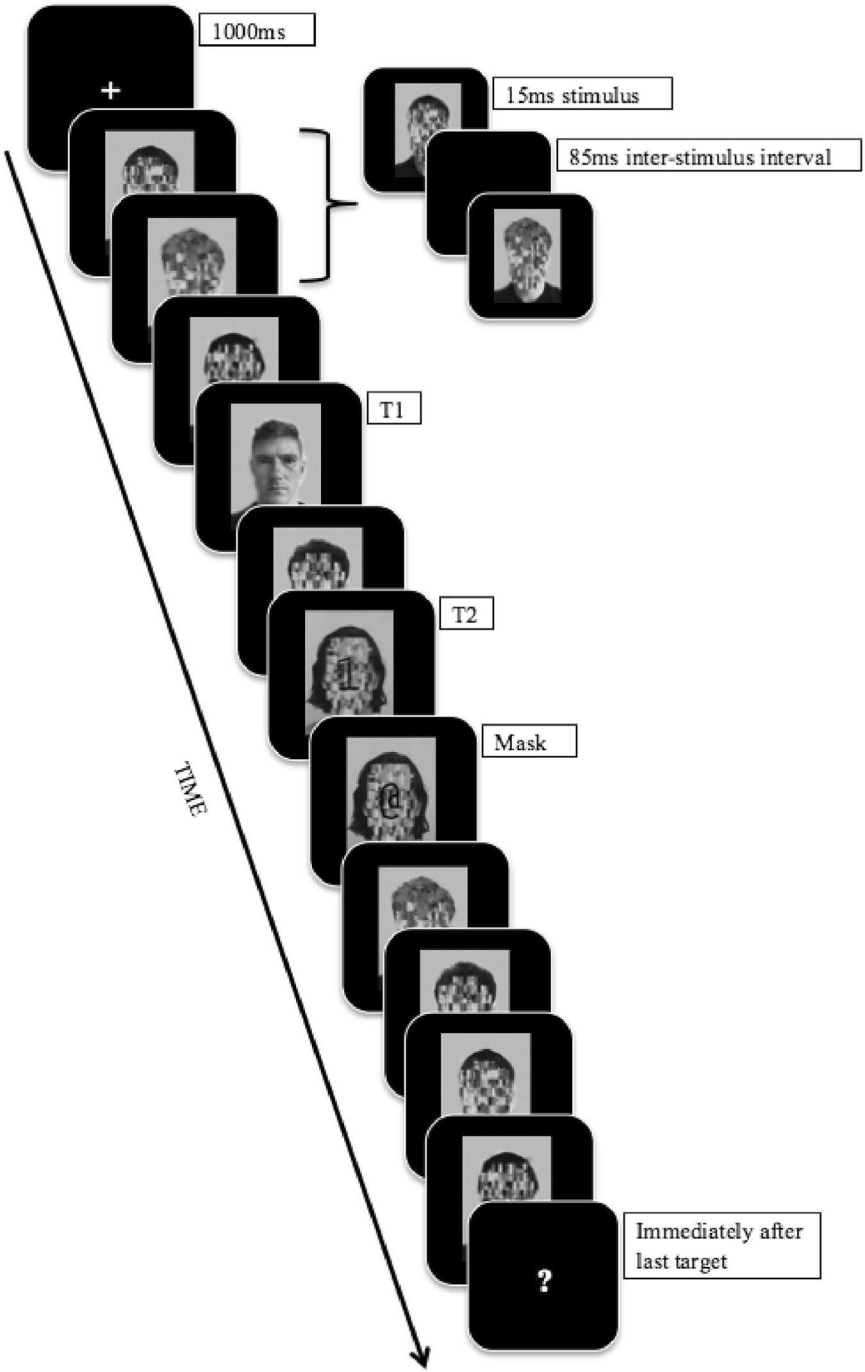
Schematic of the RSVP (lag 2 condition). Each stimuli is presented for 15 ms with an inter-stimulus interval of 85 ms – an example is presented at the top of the figure. The schematic is not to scale – stimuli are disproportionally enlarged to show detail. The figure depicts examples of the scrambled, T1 and T2 stimuli, and Mask.

The design of the experiment was identical to Experiment 2 in Roberts *et al*. with a few notable changes. First, T1 stimuli consisted of the male faces varying in facial dominance (low, neutral, and high). Both the male face stimuli and distractor stimuli were the same as Roberts *et al*. Second, T2 consisted of a digit (1 to 9) superimposed in the middle of a scrambled-face distractor (size 35, Courier New font, RGB: 0, 0, 0). The item immediately following T2 consisted of a keyboard symbol (@, #, or $) superimposed in the middle of a scrambled-face distractor (size 35, Courier New font, RGB: 0, 0, 0). Third, due to the perceptual difficulty of T2, the SOA was extended to 100 ms (15 ms stimulus, 85 ms blank ISI). Fourth, given the changes to T1 and T2 stimuli, the successive questions at the end of the sequence were changed. The first question was “Which face did you see?” and participants were asked to select from amongst pictures of a high, neutral, or low dominance male face, or a fourth option labelled “No face present” by pressing a marked key. The second question was “What number did you see?” and participants responded using the number keys on the keyboard. Finally, only one catch trial was presented – a trial with no T1. Overall, participants completed 162 trials per block – divided equally amongst T1 stimuli of each dominance level at each of the three lags (e.g. 18 trials featuring high dominance at lag 2). Participants also completed 18 catch trials (six per lag) in each block to ensure participants observed the stimuli diligently. In total, participants completed 180 trials per block, and 360 trials in total. The sequence of events of a typical trial can be seen in Fig. 5.

## Results and Discussion

Data from six participants were omitted from the main analyses. Data from three participants were omitted because their overall T1 accuracy was more than 2.5 *SD* below the mean, one participant was omitted because their overall T2 accuracy score was more than 2.5 *SD* below the mean, and two participants were omitted because their overall T1 and T2 accuracy was more than 2.5 *SD* below the mean. As a result, 38 participants were included in the final analyses (*M* = 22.55, *SD* = 7.68, 12 male, 26 female).

### T1 Accuracy

Descriptive statistics for T1 accuracy are shown in Table 4. A 3 (Dominance: high, neutral, low) × 3 (Lag: 2, 4, 8) Repeated Measures ANOVA on this data (Greenhouse-Geisser corrected) yielded a significant main effect of Dominance - *F*(1.65, 61.05) = 25.55, *p* < 0.001, *η*^2^_partial_ = 0.409, but no significant main effect of Lag – *F*(1.66, 61.20) = 0.10, *p* = 0.903, *η*^2^_partial_ = 0.003, nor a significant interaction effect – *F*(4, 148) = 2.38, *p* = 0.054, *η*^2^_partial_ = 0.060. An examination of Table 4 suggests that the main effect of Dominance stems from the fact that participants were least accurate at identifying high dominance faces compared to neutral and low dominance faces. This was supported by a Bonferroni post-hoc analysis that revealed high dominance was identified significantly less accurately than neutral dominance (*p* < 0.001, Cohen’s *D* = 0.59) and low dominance (*p* < 0.001, Cohen’s *D* = 1.00) respectively.

**Table 4:** T1 accuracy as a function of lag and dominance.

	Lag 2	Lag 4	Lag 8
	*M* (*SD*)	*M* (*SD*)	*M* (*SD*)
High Dominance	86.40 (11.13)	83.55 (11.65)	85.89 (11.27)
Neutral Dominance	90.35 (7.30)	91.88 (6.31)	90.35 (8.80)
Low Dominance	92.84 (7.06)	94.59 (5.12)	94.23 (5.61)

### T2|T1 Accuracy

**Figure 6:**
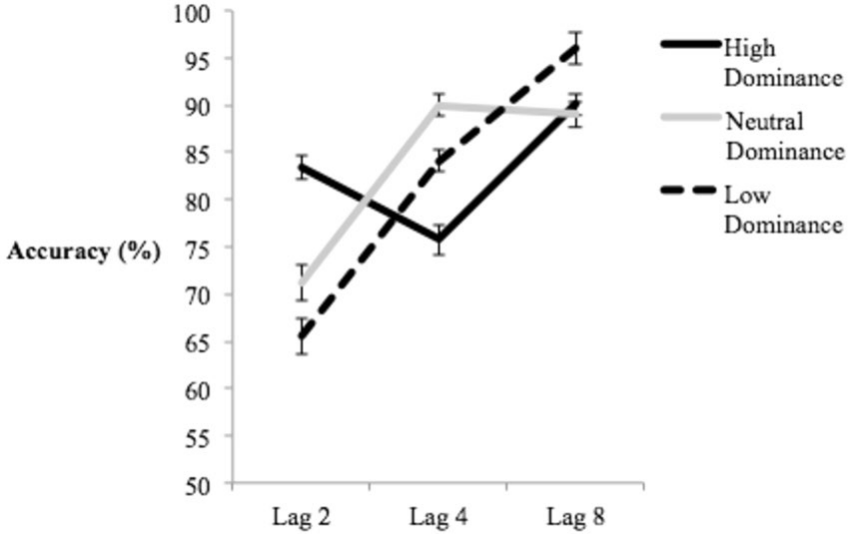
Mean accuracy of the high, low, and neutral dominance conditions across lags 2, 4, and 8. Within-subjects error bars depict 95% confidence intervals, based on O’Brien & Cousineau^79^.

Mean accuracy was calculated in the same manner as Experiment 1, and can be seen in Fig. 6. A 3 (Dominance) x 3 (Lag) Repeated Measures ANOVA (Greenhouse-Geisser corrected) yielded no main effect of Dominance, *F*(2, 74) = 1.51, *p* = 0.228, *η*^2^_partial_ = 0.039, but a significant main effect of Lag, *F*(1.46, 54.05) = 57.75, *p* < 0.001, *η*^2^_partial_ = 0.609, indicating a robust AB, and a significant interaction effect, *F*(2.48, 91.72) = 40.17, *p* < 0.001, *η*^2^_partial_ = 0.521.

To probe the significant interaction, one-way ANOVAs were conducted to compare accuracy across levels of Dominance at the two shorter lags in which maximal AB interference is expected. At Lag 2, this yielded a significant main effect – *F*(2, 74) = 39.09, *p* < 0.001, *η*^2^_partial_ = 0.514. Fisher’s LSD post-hoc analyses showed T2 accuracy was significantly poorer when T1 was a low dominance face compared to neutral dominance face (*p* = 0.007, Cohen’s *D* = 0.29). Conversely, the analyses also demonstrated accuracy was significantly greater when T1 was a high dominance face compared to when it was a neutral (*p* < 0.001, Cohen’s *D* = 0.75) and low dominance face (*p* < 0.001, Cohen’s *D* = 1.06). This is consistent with what would be expected on the basis of the competition account proposed by Roberts *et al*. (2019).

An identical one-way ANOVA at Lag 4 also yielded a statistically significant main effect, *F*(2, 74) = 27.09, *p* < 0.001, *η*^2^_partial_ = 0.423. Fisher’s LSD post-hoc analyses again showed T2 accuracy was significantly poorer when T1 was a low dominance compared to a neutral dominance face (*p* = 0.001, Cohen’s *D* = 0.56). This again replicates the low dominance effect found by Roberts *et al*. (2019). Interestingly, T2 accuracy was also significantly poorer when T1 was a high dominance face compared to neutral (*p* < 0.001, Cohen’s *D* = 1.08) or low dominance face (*p* < 0.001, Cohen’s *D* = 0.55).

Finally, an identical one-way ANOVA at Lag 8 yielded a statistically significant main effect, *F*(2, 74) = 15.99, *p* < 0.001, *η*^2^_partial_ = 0.302. Fisher’s LSD post-hoc analyses revealed significant differences only between low dominance and both high (*p* < 0.001, Cohen’s *D* = 0.84) and neutral (*p* < 0.001, Cohen’s *D* = 0.98) dominance faces. This pattern is like that seen in Experiment 1, and again may suggest low dominance faces have a perceptual advantage. However, as in earlier experiments, ceiling levels of performance indicate experimental outcomes must be interpreted cautiously.

The finding that high dominance faces impact the AB at Lag 4 is reminiscent of a similar advantage shown for T2 high dominance faces in Experiment 2 of Roberts *et al*. (2019) at early and late lags. Roberts *et al*. (2019) posited that the extremes of male dominance (low or high) appear to have a general perceptual advantage over neutral dominance faces – as indicated by greater identification at Lags 2 and 8. However the current study appears to refute this idea, since the effects were found at the nadir of the AB – that is, at the height of inter-item competition. The current study suggests, in contrast to the consistent advantage for low dominance faces over neutral dominance faces in competition for resources, that high dominance faces have a heightened ability at competing for attentional resources (i.e. Lag 4 here; Lag 2 in Roberts *et al*.) but at other times have a reduced ability (i.e. Lag 2 here). Moreover, given the consistency of findings that indicate low dominance has an advantage for attentional resources, alongside the findings of T1 that demonstrate a similar bias – low dominance stimuli may have a stimulus-driven perceptual advantage over other competing stimuli.

## General Discussion

Roberts *et al*.^34^ demonstrated that individuals with facial features associated with low dominance are more easily identified than faces associated with neutral and high dominance features when competing with other stimuli for attentional resources. The aims of the present work were to test two possible explanations for this effect. Firstly, in Experiment 1, an explanation based on the notion that counter-stereotypical stimuli attract attentional resources was tested. Then, in Experiment 2, an explanation based on the notion that low dominance stimuli have an advantage in a competition for resources over other attended stimuli was examined.

Experiment 1 tested the counter-stereotypicality account both by measuring levels of counter-stereotypicality and assessing whether high dominance female faces showed performance benefits in an AB task. Although the valence of perceptual ratings of female faces suggested high dominance female faces were perceived counter-stereotypically, there was no evidence to suggest these faces were preferentially attended. The other key finding that emerged was that the valence of perceptual ratings for male faces were not in line with the counter-stereotypicality argument, as both low and high dominance males were rated significantly negatively. Together these findings suggest that the competitive advantage seen for low dominance faces does not arise from an attentional bias towards counter-stereotypical visual stimuli.

A third, and unexpected, finding was evidence for a bias towards high dominance female faces, not seen with male faces, possibly indicating that the dominance pathway to social status may differ for males and females. Social status theorists agree that the dominance pathway has readily physical discernable features, generally associated with heightened masculinity^5,6,27^. In the absence of such physical dominance cues, it may be the case that different facial features and/or that different perceived attributes, such as trustworthiness or attractiveness, may preferentially attract attention^26,57^. When examining attraction towards faces, the features that draw attention differ depending on the sex of the perceived individual. For females, features include eye size^59^ and features affected by oestrogen^60^, whereas in males, typically it is features that are affected by testosterone that attract attention (see Thornhill & Gangestad^60^ for a review). It may also differ based on traits of the perceiver rather than the individual being perceived. A study by Mileva, Jones, Russell, and Little^61^ showed that when viewing females wearing cosmetic products, men were more likely to rate the faces higher in prestige-based status and women were more likely to rate the faces higher in dominance-based status. It is unclear from the current study whether the facial features in male dominance translate to female dominance, and further research is needed to understand the features and effects of female dominance-based status.

Experiment 2 tested the attention competition account – that low dominance faces have a greater ability to compete for attentional resources. This account was tested using a modified AB task, in which T2 stimuli (digits superimposed in the middle of scrambled face distractors) had to break the through the attentional competition posed by male facial dominance stimuli as T1. As previously discussed, the bias of low dominance stimuli was supported, as T2 stimuli that followed low dominance faces were identified consistently less accurately than neutral dominance stimuli – supporting the findings of Roberts *et al*. Moreover, the results indicate that on certain occasions high dominance had a reduced ability to bias attention but at other times demonstrated a heightened ability at biasing attention.

The findings of the current study provide further evidence that low dominance male faces have an advantage when competing for attentional resources. Of particular interest, therefore, is to understand which features of low dominance stimuli faces make them attentionally salient. T1 data in Experiment 2 showed that, even though identification was above 80% accuracy, low dominance faces were identified significantly better than neutral and high dominance faces. As such, one possibility is that low dominance faces have a perceptual advantage over neutral and high dominance faces in a stimulus driven process compared to a cognitive driven process^62^. For example, recent evidence suggests that the direction of a head tilt of a neutral dominance face can increase perceptions of dominance^63^.

A method that could be used to explore whether low dominance faces have a stimulus-driven perceptual advantage is to use electroencephalography (EEG) to conduct a Fast Periodic Visual Stimulation (FPVS) study. In FPVS paradigms, a stimulus is repeated at a periodic rate to elicit synchronisation of the brain to the same frequency^64–66^ Importantly, FPVS paradigms have been used to explore perceptual advantages towards pre-conscious identification of faces^67–69^ – see Rossion^66^. For example, Rossion *et al*.^69^ found that if a face was placed at alternation frequencies, the brain showed greater activation at those synchronised frequencies. Specifically – Rossion *et al*. demonstrated that faces at alternation frequencies of 1.18 hz (within a 5.88 hz stream of images) yielded significant 1.18 hz oscillations in neural responses. This effect was not noted for low contrast faces or objects placed at the same alternation frequencies, which indicates the brain has specific perceptual biases to face identification. Moreover, this paradigm could be used to examine perceptual biases towards low dominance faces compared to neutral and high dominance faces – by measuring the differences in neural oscillation magnitudes at alternate frequencies for faces varying in dominance. Using the FPVS paradigm would be a useful tool for exploring whether the low dominance attention bias is a stimulus-driven process.

A different approach to this question could be through the use of inverted faces. This technique has a long history in face perception research^70–72^, with many studies indicating that inverting faces impairs multiple face processing abilities including face matching and identification of newly learned faces^73–76^. Such impairments, in turn, suggest that the advantage of viewing an upright face is the ability to differentiate and identify higher-order facial information. By comparison, individual features of faces are more easily attended to in inverted faces^77–79^. This suggests that people have the ability to differentiate physical indicators of inverted faces compared to other objects, despite reduced ability to identify higher-order facial information. As such, an inverted face paradigm could be used to explore whether the low dominance effect occurs because of sensitivity to a specific facial feature (e.g. lower acuity signals such as a wider jaw vs. masculinised jaw) or whether the effect is a result of identifying facial dominance. For example, if the low dominance advantage was due to perceptual indicators (such as low-level features), inverted low dominance face stimuli should show a consistent bias of attention. However if the bias is due to cognitive indicators, then only upright faces should show the advantage. On the basis of the data presented here these explanations are merely speculative, however they make distinctly different and testable predictions.

A final question that arose in this study, in relation to the findings of Roberts *et al*.^34^, is the variability of high dominance on performance. As previously discussed, Experiment 2 found that high dominance varies between having a reduced and a heightened ability at biasing attention. When comparing the non-spatial attention effects of high dominance between Roberts *et al*. and the current study, it appears that high dominance does not have a consistent attentional bias. Further research is needed to clarify the exact nature of non-spatial attention towards high dominance, and how this connects with current understandings gaze and social attention^14,29^.

In conclusion, the study tested two explanations that could explain the low dominance effect. The first account of counter-stereotypicality was not supported as non-spatial attention was biased towards both high and low dominance female faces. The second account, attention competition, was supported – finding that low dominance faces had a consistent advantage at competing for attention resources. These findings add to the social status literature in evolutionary, cognitive, and social psychology – by challenging our current understanding of how humans interact with status. They also suggest a pathway to future research; examining the feature(s) of low dominance male stimuli that make them attentionally salient.
