# Publisher Correction: Unravelling how low dominance in faces biases non-spatial attention

**DOI:** 10.1038/s41598-020-57849-3

**Published:** 2020-01-21

**Authors:** Ashton Roberts, Romina Palermo, Troy A. W. Visser

**Affiliations:** 1 School of Psychological Science, The University of Western Australia, Perth, Western Australia Australia; 2 ARC Centre of Excellence in Cognition and its Disorders (CCD), Sydney, Australia

Correction to: *Scientific Reports* 10.1038/s41598-019-54295-8, published online 29 November 2019

The original version of this Article contained a typographical error in the Abstract.

“According to the Dual Dodel of Social Hierarchy, one pathway for attaining social status is through dominance (coercion and intimidation).”

now reads:

“According to the Dual Model of Social Hierarchy, one pathway for attaining social status is through dominance (coercion and intimidation).”

This error has now been corrected in the PDF and HTML versions of the Article.

